# Trajectories of antenatal depression and adverse pregnancy outcomes: A prospective cohort study

**DOI:** 10.1192/j.eurpsy.2025.10058

**Published:** 2025-07-21

**Authors:** Jianfei Chen, Siqi Li, Sai Yang, Yang Chen, Jackson Jr Nforbewing Ndenkeh, Intan Maharani Sulistyawati Batubara, Luyang Zhu, Jun Liu, Xinlong Pan, Zhijie Zou, Cuifang Fan, Xiaoli Chen, Zhao Ni

**Affiliations:** 1 School of Nursing, Wuhan University, Wuhan, China; 2 Department of Nursing, Union Hospital, Tongji Medical College, Huazhong University of Science and Technology, Wuhan, China; 3 School of Nursing, Yale University, New Haven, CT, USA; 4 Department of Obstetrics, Renmin Hospital of Wuhan University, Wuhan, China

**Keywords:** adverse pregnancy outcomes, antenatal depression, risk factors, trajectories, cohort study

## Abstract

**Background:**

Antenatal depression symptom is a global health concern, but the trajectories of antenatal depression symptom vary across different studies. Additionally, the influencing factors and adverse pregnancy outcomes of antenatal depression symptom may differ across heterogeneous subtypes, which requires further exploration.

**Methods:**

A prospective cohort study was conducted in Hubei province, China, from July 2022 to September 2023. Pregnant women (<14 weeks) were enrolled and followed up at 16, 21, 28, and 37 gestational weeks, with depressive symptom measured using the Edinburgh Postnatal Depression Scale (EPDS). Latent class growth modeling and logistic regression were used for data analysis.

**Results:**

Of 1034 women enrolled, 725 completed all follow-ups. Four depressive symptom trajectories were identified: no depression group (32.13%), persistent subclinical depression group (42.48%), persistent moderate depression group (19.17%), and persistent high depression group (6.21%). Risk factors of depressive symptom trajectories included low social capital, unplanned pregnancy, primiparity, mental illness history, high perceived stress, and low resilience (*p* < 0.05). Compared to the no depression group, gestational diabetes mellitus (GDM) risk was 1.90 times higher in the persistent moderate group and 2.59 times higher in the persistent high group; small for gestational age (SGA) risk was 2.42 times higher in the persistent moderate group and 3.98 times higher in the persistent high group.

**Conclusions:**

This study identified four antenatal depressive symptom trajectories. Persistent moderate and high depression groups were linked to GDM and SGA, highlighting the importance of mental health assessments and intervention for pregnant women, especially those with higher depression severity, to prevent adverse outcomes.

## Introduction

Antenatal depressive symptoms refer to persistent feelings of sadness, anxiety, and a lack of interest in daily activities during pregnancy [1]. It is estimated that 15–65% of women worldwide are affected by antenatal depressive symptoms [2], with a prevalence of 19.7% in China [3]. These symptoms have a profound impact on the development and health of the offspring and require timely intervention [4]. However, the entire course of antenatal depressive symptoms remains unclear. Previous research on the trajectory of prenatal depression symptoms is limited, and its findings are inconsistent, which complicates screening and referral [5]. Adverse pregnancy outcomes associated with antenatal depression symptoms include hypertensive disorders complicating pregnancy (HDCP), gestational diabetes mellitus (GDM), placental abruption, cesarean section, preterm birth (PTB), fetal growth restriction, small for gestational age (SGA), and low Apgar scores [6–8]. Although the link between antenatal depressive symptoms and these adverse outcomes has been well established, depressive symptom trajectories, rather than simple depression screening, may provide a better understanding of the underlying physiological dysregulation. Given that evolving pathophysiology could contribute to an increased risk of adverse pregnancy outcomes, analyzing depressive symptom trajectories offers a more biologically relevant framework for understanding these risks [9]. Considering the availability of data, this study focuses on HDCP, PTB, GDM, and SGA as the primary outcomes of interest. HDCP and GDM are common pregnancy complications that pose significant risks to maternal health and can lead to adverse fetal outcomes [10, 11]. Additionally, PTB and SGA, which are directly associated with neonatal health, have also been linked to antenatal depressive symptoms [12, 13]. These outcomes cover various dimensions of maternal and infant health and reflect the complex interplay of physiological and psychological changes. Understanding the influencing factors of the trajectories is equally important. Early intervention targeting measurable high-risk factors could prevent the onset of antenatal depression and mitigate adverse outcomes. Prior research has underscored the roles of various physiological, psychological, and social factors in shaping antenatal depressive symptoms [14]. For instance, a history of depression, income levels, and anxiety have been shown to affect the trajectory of antenatal depression [15, 16]. Furthermore, low psychological resilience, limited social capital, and high perceived stress have been identified as risk factors for the development of antenatal depression in cross-sectional studies [17]. However, the relationship between these influencing factors and antenatal depression trajectories remains insufficiently explored, and further research is needed to investigate how these factors contribute to the development and progression of antenatal depression.

Given the need for a deeper understanding of antenatal depressive symptoms, this study has three main objectives: (1) to explore the trajectories of antenatal depressive symptoms from early pregnancy to predelivery; (2) to investigate the associated influencing factors; and (3) to examine the relationship between these trajectories and adverse pregnancy outcomes. By identifying specific patterns of depressive symptom trajectories and correlating them with risk factors and adverse outcomes, this study aims to provide valuable insights into the dynamic characteristics of antenatal depression and its implications for clinical practice.

## Materials and methods

### Study design

This study was a part of a maternity cohort study conducted at a tertiary hospital in Wuhan, Hubei Province (Project No. 21BSH073). Eligible pregnant women (*N* = 1034) were enrolled from early pregnancy (T0) from Renmin Hospital of Wuhan University’s obstetrics clinic between July 2022 and September 2023 [18]. After completing a baseline assessment, participants were followed up during the second trimester (16–20 weeks, T1), early third trimester (21–24 weeks, T2), and late third trimester (28–36 weeks, T3 and 37–40 weeks, T4).

The inclusion criteria were: (1) women aged 18–50 years, (2) in early pregnancy (gestational age <14 weeks), (3) carrying a singleton pregnancy, (4) planning to receive regular prenatal care and deliver at the hospital; (5) having completed the baseline assessments and all four follow-up visits. Exclusion criteria included: (1) diagnosed with severe cardiovascular, neurological, or renal diseases, (2) inability to read or write, impairing questionnaire completion, (3) lack of access to a smartphone or the Internet.

The study protocol was approved by the Ethics Committee of Wuhan University (WHU2021-YF001). Informed consent was obtained from each study participant.

### Sample size

The required sample size was calculated using the formula [19]: 
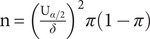
 , where the significance level *α* = 0.05 was set, the margin of error *δ* = 0.04, and *π* represents the estimated prevalence of antenatal depression. Based on a previous meta-analysis reporting a prevalence of 19.7% for antenatal depression [3], and allowing for a 4% margin error the initial sample size was calculated to be 380. To account for an anticipated dropout rate of 15% over the course of the study, we aimed to recruit approximately 450 participants to ensure sufficient data for robust analysis.

### Study process

Data were collected at baseline (T0) and during follow-up visits scheduled in the second trimester (16–20 weeks, T1), early third trimester (21–24 weeks, T2), and late third trimester (28–36 weeks, T3 and 37–40 weeks, T4). Detailed data collection procedures are described in our previous protocol [18].

General information, including age, household characteristics, marital status, education level, and household income, along with obstetric variables such as pre-pregnancy BMI, parity, number of pregnancies, adverse maternity history, planned pregnancy, method of conception, history of mental illness, were assessed at baseline using a self-designed questionnaire.

At each follow-up, participants completed a series of electronic questionnaires including the EPDS, the Personal Social Capital Scale-16 [20], the Perceived Stress Scale-14 [21, 22], and the Connor–Davidson Resilience Scale-10 [23]. These variables were examined as covariates in relation to pregnancy outcomes. Medical and obstetric data were extracted from the electronic medical records, 1 week after delivery to assess pregnancy outcomes. The delivery complications studied included GDM, HDCP, PTB, and SGA (for detailed definitions of these outcomes, see Supplementary Text 1).

### Depression evaluations

The Edinburgh Postnatal Depression Scale (EPDS) is the most widely used self-assessment screening tool for measuring antenatal depression. Developed by Cox in 1987 [24], this scale was first translated into Chinese by Peng et al. in 1994 [25]. In 1998, Li et al. validated the Chinese version of the EPDS among pregnant women in China, demonstrating that the scale possesses good reliability and validity within this population [26]. The EPDS comprises 10 items, each scored from 0 (no symptoms) to 3 (severe symptoms), resulting in a total possible score ranging from 0 to 30; higher scores indicate more severe depressive symptoms. The cutoff values for identifying potential depressive symptoms vary from 9 to 13, depending on the country, setting, and cultural background of the study [27–29]. Among them, the expert consensus in China recommends a cutoff value of 9 [29]. In this study, the Cronbach’s alpha coefficients for the EPDS at five time points were 0.794, 0.853, 0.846, 0.839, and 0.884.

### Statistical analysis

Data were entered using EpiData and analyzed using SPSS version 29.0 and Mplus version 8.0.

Data analysis consisted of four stages. First, descriptive statistics were calculated, reporting continuous variables as means and standard deviations (SD) and categorical variables as frequencies and percentages (%). Subsequently, latent class growth modeling (LCGM) was then employed to identify heterogeneous developmental trajectories of antenatal depression, utilizing model fit indices such as the Akaike information criterion (AIC), Bayesian information criterion (BIC), adjusted BIC, entropy, the Lo–Mendell–Rubin adjusted likelihood ratio test (LMRLRT), and bootstrapped likelihood ratio test (BLRT).

Third, differences in physiological, psychological, and social factors influencing the latent categories of antenatal depression trajectories are analyzed using ANOVA, chi-square tests, and Fisher’s exact test. Multinomial logistic regression is used to investigate the influencing factors. The dependent variable is the developmental trajectory of prenatal depressive symptoms, with the low symptom group as the reference category. Variables with *p* < 0.1 in the univariate analysis are included in the multinomial logistic regression to explore the factors influencing the other groups compared to the reference group. This is a common practice used to avoid excluding potentially important predictors at an early stage of data analysis [30].

Finally, binary logistic regression was used to examine association between pregnancy outcomes and the latent categories of antenatal depression trajectories. Two models were employed: Model 1 included only the latent categories of antenatal depression as independent variables, while Model 2 controlled for confounding factors by adjusting for baseline characteristics identified as significant in univariate analysis (*p* < 0.1). The dependent variable was the occurrence of pregnancy outcomes (e.g., GDM), with the low symptom group serving as the reference category.

## Results

### Baseline characteristics of study participants

**Figure 1.**
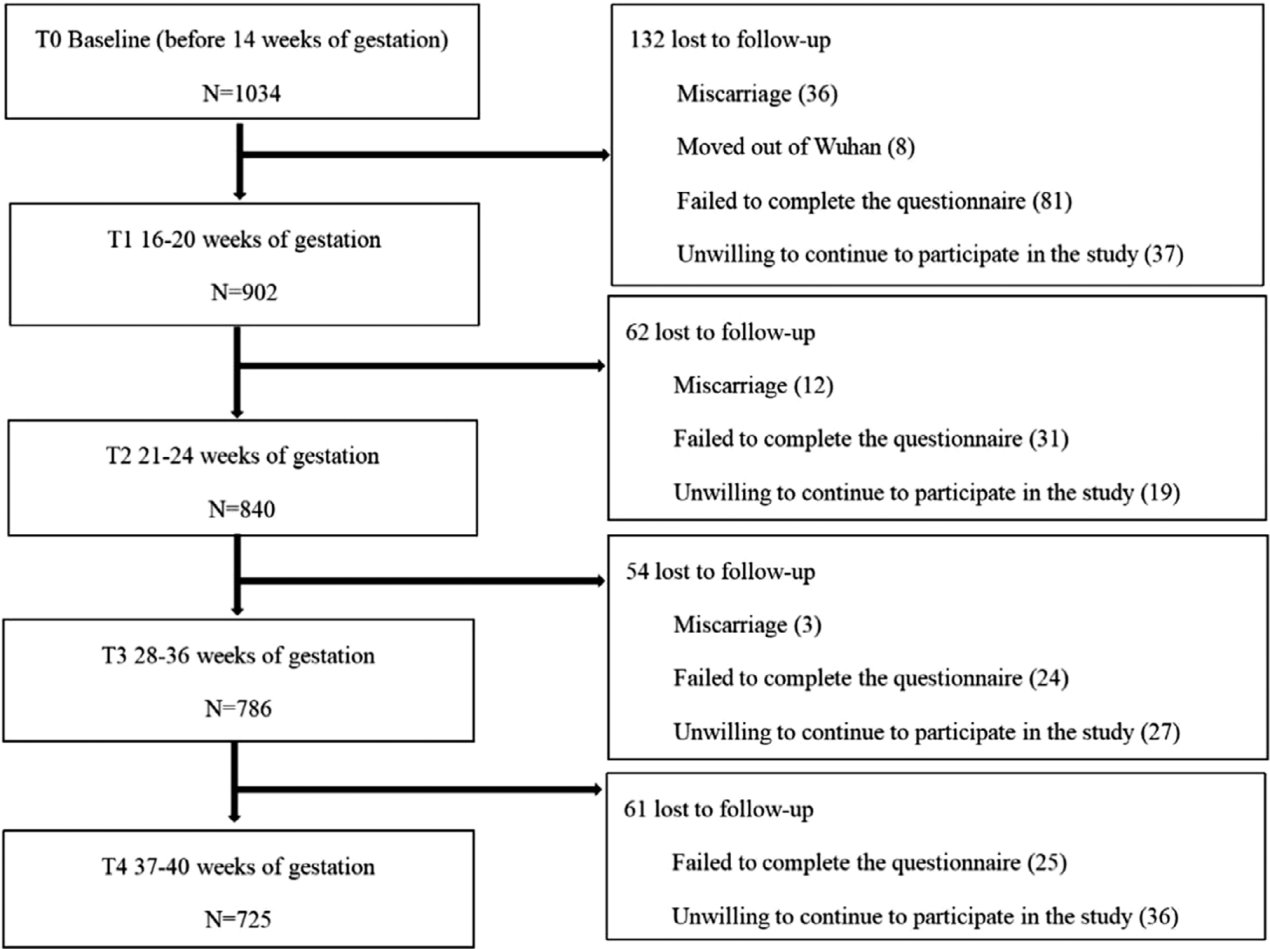
Flow chart of study participants.

In this study, 1034 pregnant women were included at baseline (T0), the final sample size for the analysis consisted of 725 participants (Figure 1). Participants who failed to complete the questionnaire at any of the four follow-up points were excluded from the study to ensure the integrity and consistency of the longitudinal data. Table 1 presents the baseline characteristics of retained participants and those lost to follow-up. The results showed that there were statistically significant differences between the two groups in terms of age, social capital, income, and whether they were planning to get pregnant.

**Table 1.** Baseline characteristics and comparison of retained and lost to follow-up individuals

Item		Retained(*n* = 725)	Lost to follow-up(n = 309)	*p*
Age, years		32.62 ± 3.72	32.23 ± 4.06	0.035*
EPDS (T0)		8.64 ± 3.55	9.08 ± 3.85	0.108
Social capital score		46.86 ± 8.24	45.81 ± 8.52	0.020*
Household	Urban	411 (56.7%)	179 (57.9%)	0.719
	Rural	314 (43.3%)	130 (42.1%)	
Marriage	Married	699 (96.4%)	296 (95.8%)	0.764
	Unmarried	26 (3.6%)	13 (4.2%)	
Education level	≤High school	102 (14.1%)	22 (7.1%)	0.100
	Junior college	187 (25.8%)	109 (35.3%)	
	Undergraduate	316 (43.5%)	141 (45.6%)	
	≥Masters	120 (16.6%)	37 (12.0%)	
Household income, RMB	≤5000	50 (6.90%)	26 (8.4%)	0.016*
	5000–10000	226 (31.17%)	97 (31.4%)	
	10001–20000	300 (41.38%)	150 (48.5%)	
	≥20000	149 (20.55%)	36 (11.7%)	
Pre-pregnancy BMI, kg/m^2^		21.80 ± 3.05	21.66 ± 3.05	0.353
Parity	Primigravida	544 (75.0%)	221 (71.5%)	0.314
	Menstruation	181 (25.0%)	88 (28.5%)	
Number of pregnancies	Once	397 (54.7%)	151 (48.9%)	0.134
	Twice	210 (29.0%)	92 (29.8%)	
	≥3 times	118 (16.3%)	66 (21.3%)	
Adverse maternity history	Yes	221 (30.5%)	103 (33.3%)	0.444
	No	504 (69.5%)	206 (66.7%)	
Planned pregnancy	Yes	566 (78.1%)	216 (66.9%)	0.002*
	No	159 (21.9%)	93 (30.1%)	
Method of conception	Natural pregnancy	613 (84.6%)	276 (89.3%)	0.053
	Artificial assisted reproduction	112 (15.4%)	33 (10.7%)	
History of mental illness	Yes	46 (6.3%)	23 (7.4%)	0.494
	No	679 (93.7%)	286 (92.6%)	

*Note:* The significant (i.e., *p*<0.05) *p* values are shown in bold.

The average age of retained participants was 32.62 ± 3.72 years, with a distribution of 56.7% urban and 43.3% rural residents. Education levels were as follows: 14.1% had completed high school or lower, 25.8% held a junior college degree, 43.5% had a bachelor’s degree, and 16.6% had obtained a postgraduate degree. The average pre-pregnancy BMI was 21.80 ± 3.05 kg/m^2^. Only 6.3% (46) reported a previous history of mental illness and 1.1% (8) had used psychiatric medications before pregnancy.

### Trajectories of antenatal depression

**Figure 2.**
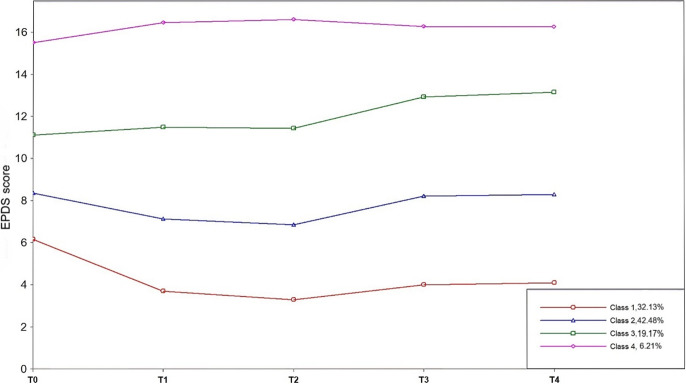
Trajectory of antenatal depressive symptom development. *Note*: Class 1, no depression group (32.13%); Class 2, persistent subclinical depression group (42.48%); Class 3, persistent moderate depression group (19.17%); Class 4, persistent high depression group (6.21%).

This study employed the LCGM to explore trajectory models ranging from one to six classes. Fit parameters for each model are presented in Supplementary Table S1, while the distribution of individuals across each latent class is detailed in Supplementary Table S2. The results indicate that as the number of latent classes increases, the AIC, BIC, and adjusted BIC values decrease, signifying improved model fit. Entropy values were greater than 0.8 for models with two to four latent classes but dropped below 0.8 for the fifth and sixth classes, suggesting that the optimal model should compromise two or four classes. The LMRLRT and BLRT results yielded significant *p* values (<0.001) for models with two to four latent classes, indicating that the three-class model fits better than the two-class model and that the four-class model fits better than the three-class model. Consequently, this study selected a four-class trajectory model (Figure 2).

The parameters for the latent class growth model of antenatal depression trajectory subgroups are summarized in Supplementary Table S3. Based on the expert consensus in China recommending a cutoff value of 9, as well as the shape and levels of the trajectories shown in Figure 2, the subgroups were defined as follows: no depression group (233 individuals); persistent subclinical depression group (308 individual); persistent moderate depression group (139 individuals); and persistent high depression group (45 individuals).

A repeated measures ANOVA on EPDS scores across the four groups and five time points revealed significant time and class effects, as well as a significant interaction between time and class, indicating varying changes in EPDS scores across the trajectories. Detailed scores and prevalence can be found in Supplementary Table S4, and the model validation results are in Supplementary Table S5.

### Risk factors of depression trajectories

Univariate analysis identified several factors influencing the trajectory of prenatal depressive symptom (*p* < 0.1), including social capital (*p* < 0.001), planned pregnancy (*p* < 0.002), pre-pregnancy BMI (*p*: 0.073), parity (*p* < 0.080), history of mental illness (*p* < 0.001), perceived stress (*p* < 0.001), and psychological resilience (*p* < 0.001). Detailed results are presented in Supplementary Table S6.

Subsequently, the statistically significant factors (*p* < 0.1) influencing the trajectory of prenatal depressive symptoms were included in the multinomial logistic regression analysis. The results indicated that compared to the no depression group, the factors influencing the persistent subclinical depression group (*p* < 0.05) included unplanned pregnancy (OR: 2.650, 95% CI: 1.562–4.497), social capital (OR: 0.962, 95% CI: 0.938–0.985), psychological resilience (OR: 0.911, 95% CI: 0.881–0.942), and perceived stress (OR: 1.091, 95% CI: 1.066–1.117). For the persistent moderate depression group (*p* < 0.05), the influencing factors were unplanned pregnancy (OR: 2.581, 95% CI: 1.363–4.887), history of mental illness (OR: 8.692, 95% CI: 2.739–27.588), social capital (OR: 0.941, 95% CI: 0.912–0.971), psychological resilience (OR: 0.838, 95% CI: 0.802–0.875), and perceived stress (OR: 1.094, 95% CI: 1.061–1.128). In the persistent high depression group (*p* < 0.05), the significant factors included primiparity (OR: 4.773, 95% CI: 1.506–15.126), unplanned pregnancy (OR: 5.443, 95% CI: 2.289–12.939), social capital (OR: 0.907, 95% CI: 0.866–0.950), psychological resilience (OR: 0.823, 95% CI: 0.771–0.879), and perceived stress (OR: 1.209, 95% CI: 1.147–1.274). Detailed results are presented in Table 2.

**Table 2.** Multinomial logistic regression analysis of factors influencing the prenatal depressive symptom development trajectory (*N* = 725)

	Persistent subclinical depression group			Persistent moderate depression group			Persistent high depression group		
	*B*	OR (95% CI)	*p*	*B*	OR (95% CI)	*p*	*B*	OR (95% CI)	*p*
Parity									
Primipara vs. Multipara	0.149	1.161 (0.740–1.820)	0.515	0.078	1.081 (0.611–1.915)	0.788	1.563	4.773 (1.506–15.126)	0.008
Planned pregnancy									
Yes vs. no	0.975	2.650 (1.562–4.497)	<0.001	0.948	2.581 (1.363–4.887)	0.004	1.694	5.443 (2.289–12.939)	<0.001
History of mental illness									
Yes vs. no	0.602	1.825 (0.564–5.912)	0.316	2.162	8.692 (2.739–27.588)	<0.001	1.484	4.409 (0.984–19.753)	0.051
Pre-pregnancy BMI	0.130	1.013 (0.951–1.079)	0.684	−0.085	0.919 (0.844–1.001)	0.051	−0.033	0.968 (0.856–1.094)	0.602
Social capital	−0.039	0.962 (0.938–0.985)	0.002	−0.061	0.941 (0.912–0.971)	<0.001	−0.098	0.907 (0.866–0.950)	<0.001
Resilience	−0.093	0.911 (0.881–0.942)	<0.001	−0.177	0.838 (0.802–0.875)	<0.001	−0.194	0.823 (0.771–0.879)	<0.001
Perceived stress	0.088	1.091 (1.066–1.117)	<0.001	0.090	1.094 (1.061–1.128)	<0.001	0.190	1.209 (1.147–1.274)	<0.001

### Association between antenatal depression trajectories and perinatal outcomes

Univariate analysis results for GDM, HDCP, PTB, SGA, and baseline characteristics are shown in Supplementary Tables S7–S10, respectively.

Binary logistic regression for GDM showed that: In Model 1, compared to the no depression group, the persistent moderate depression group (OR: 2.040, 95% CI: 1.199–3.470, *p*: 0.009) and the persistent high depression group (OR: 3.030, 95% CI: 1.474–6.231, *p*: 0.003) were significantly associated with GDM. In Model 2, the persistent moderate depression group (OR: 1.899, 95% CI: 1.031–3.497, *p*: 0.040) and the persistent high depression group (OR: 2.585, 95% CI: 1.135–5.884, *p*: 0.024) remained significantly associated with GDM (Table 3).

**Table 3.** Binary logistic regression analysis of trajectories of antenatal depressive symptoms and adverse pregnancy outcomes (*N* = 725)

		Persistent subclinical depression group		Persistent moderate depression group		Persistent high depression group	
		OR (95% CI)	*p*	OR (95% CI)	*p*	OR (95% CI)	*p*
Gestational diabetes	Model 1	1.558 (0.983–2.470)	0.059	2.040 (1.199–3.470)	0.009	3.030 (1.474–6.231)	0.003
	Model 2	1.387 (0.834–2.306)	0.207	1.899 (1.031–3.497)	0.040	2.585 (1.135–5.884)	0.024
Gestational hypertension	Model 1	0.658 (0.334–1.296)	0.226	0.780 (0.343–1.775)	0.553	-	-
	Model 2	0.918 (0.418–2.015)	0.831	1.062 (0.399–2.826)	0.904	-	-
Preterm birth	Model 1	1.233 (0.758–2.004)	0.399	1.292 (0.719–2.321)	0.391	1.629 (0.716–3.708)	0.245
	Model 2	1.260 (0.728–2.181)	0.409	1.429 (0.722–2.828)	0.306	2.057 (0.807–5.246)	0.131
Small for gestational age	Model 1	1.442 (0.736–2.828)	0.286	2.327 (1.118–4.843)	0.024	3.911 (1.576–9.701)	0.003
	Model 2	1.424 (0.686–2.956)	0.343	2.422 (1.059–5.537)	0.036	3.982 (1.393–11.379)	0.010

*Note*: Model 1: Independent variables are different potential categories of antenatal depressive symptom development trajectories.

Model 2:Independent variables are different potential categories of antenatal depressive symptom development trajectories, controlling for statistically significant baseline characteristics in one-way analyses (gestational diabetes: controlling for social capital, pre-pregnancy BMI, method of conception, resilience; gestational hypertension: controlling for social capital, pre-pregnancy BMI, perceived stress; preterm birth: controlling for age, social capital, pre-pregnancy BMI, parity, number of pregnancies; small for gestational age: controlling for resilience, number of pregnancies).

Binary logistic regression for SGA showed that: In Model 1, the persistent moderate depression group (OR: 2.327, 95% CI: 1.118–4.843, *p*: 0.024) and the persistent high depression group (OR: 3.911, 95% CI: 1.576–9.701, *p*: 0.003) were significantly associated with SGA compared to the low symptom group. In Model 2, these associations remained significant: persistent moderate depression group (OR: 2.422, 95% CI: 1.059–5.537, *p*: 0.036) and persistent high depression group (OR: 3.982, 95% CI: 1.393–11.379, *p*: 0.010) (Table 3).

In both Model 1 and Model 2, the persistent subclinical, moderate, and high depression groups were not significantly associated with HDCP and PTB when compared to the no depression group (Table 3).

## Discussion

### Main findings

In this prospective cohort study, we explored the relationship between the latent categories of antenatal depression symptom trajectories, various risk factors, and four adverse pregnancy outcomes (GDM, HDCP, SGA, and PTB). We identified four distinct trajectories of antenatal depressive symptoms. Our findings indicated that unplanned pregnancy, low social capital, low psychological resilience, history of mental illness, multiparity, and high perceived stress were significantly associated with different trajectory groups. Regarding adverse outcomes, women in persistent moderate and high depression groups during pregnancy demonstrated an increased risk for GDM and SGA. However, no significant associations were found between any depression groups and the risk of HDCP or PTB.

#### Trajectories of antenatal depressive symptoms

This study identified four distinct trajectories of antenatal depressive symptoms: no depression group (32.13%), persistent subclinical depression group (42.48%), persistent moderate depression group (19.17%), and persistent high depression group (6.22%). By following women through five time points (T0–T4), we found that the trajectories of antenatal depression were relatively stable over time. Our results are similar to previous studies by Lim et al. and Kee et al. [31, 32]. This suggests that for many women, depressive symptoms may remain constant throughout pregnancy. These findings highlight the importance of early detection and intervention, which can help mitigate the risk of adverse pregnancy outcomes.

However, Denckla et al.’s study [33] contrasts with this, identifying four unique trajectories, including two (emergent and chronic) that exhibit instability. And this variability highlights that some women experience significant fluctuations in depressive symptoms during pregnancy. Plausible explanations for this discrepancy could include differences in the study populations or settings (e.g., England vs. China) and/or differences in the timing of the studies (e.g., 1991–95 vs. 2010), which may have contributed to variations in findings. These findings underscore the complexity of antenatal depression symptom and highlight the need for personalized monitoring and treatment.

#### Influencing factors

This study sheds light on the multifaceted relationships between antenatal depressive symptoms trajectories and various factors. From a physiological perspective, a history of mental illness was prominent in the moderate depression group, while primiparity was associated with the persistent high depression group, reflecting similar findings by Huang et al. [5]. Primiparous women may face additional stressors due to lack of knowledge and experience with pregnancy, while those with a history of mental illness may be predisposed to recurrence [34].

Psychologically, the persistent subclinical, moderate, and high depression groups exhibited higher levels of perceived stress and lower psychological resilience compared to the low depression group. These factors may arise from the multiple stressors women face during pregnancy, including concerns about childbirth, safety issues, fetal health, and postpartum recovery [35]. When these stressors exceed an individual’s coping capacity, psychological resilience may decline, leading to depressive symptoms [36]. Sociologically, unplanned pregnancy and lower social capital were associated with the persistent subclinical, moderate, and high depression groups. Unplanned pregnancy may leave women less prepared emotionally, socially, and psychologically, exacerbating the challenges of balancing parenting responsibilities and professional life [37]. Additionally, research indicates that women with unplanned pregnancy often experience lower relationship satisfaction and greater difficulty managing pregnancy-related demands [38].

#### Adverse outcomes

Our study found that compared to the no depression group, GDM risk was 1.90 times higher in the persistent moderate group and 2.59 times higher in the persistent high depression group, the significant association are consistent with previous cross-sectional studies [37, 39]. A 2020 review by Riggin et al. revealed a complex, bidirectional relationship between GDM and antenatal depression, mediated by pharmacological and psychosocial factors [40]. Furthermore, women with GDM who experience antenatal depressive symptoms tend to engage in less self-care, potentially worsening their condition [41]. This highlights the importance of addressing both mental and physical health during pregnancy to reduce the negative impact of antenatal depression and GDM on maternal health.

SGA risk was 2.42 times higher in the persistent moderate group and 3.98 times higher in the persistent high group. This contracts with Miller et al. who found no association between prenatal depression trajectories and SGA [42]. Differences in sample demographics, assessment timing, and depression classification methods may account for these discrepancies. Our study was based on a Chinese cohort, whereas Miller et al’ s study was focused on a U.S cohort. Additionally, Miller’s study only tracked depression symptoms at two points and categorized women into three groups based on changes in EPDS scores, rather than using latent class analysis. This distinction highlights the need for further research with diverse populations and refined analytic approaches to understand the nuances of antenatal depression trajectories and their impact on pregnancy outcomes.

### Clinical and research implications

These findings indicate that integrating social determinants into routine prenatal assessments is essential for identifying women at risk of developing depressive symptoms. Factors such as unplanned pregnancy can lead to emotional and financial instability, while low social capital may result in feelings of isolation and increased stress [43]. Interventions to strengthen social support networks, such as peer support groups, and community resources are vital for mitigating depressive symptoms. By acknowledging that social contexts contribute to antenatal depression, healthcare providers can create holistic strategies that address both the mental and social well-being of pregnant women, going beyond medical treatment alone.

Additionally, tailored intervention should address the severity of depressive symptoms. For instance, women in the persistent moderate depression group may benefit from regular psychological counseling and stress management programs, while those in the persistent high depression group might require more intensive mental health interventions, potentially involving pharmacological treatments and multidisciplinary care involving mental health professionals, obstetricians, and social workers. Furthermore, continuous monitoring throughout pregnancy is essential to mitigate risks associated with GDM and SGA, ensuring timely interventions that optimize maternal and neonatal health outcomes.

### Strengths and limitations

The strengths of this study are as follows. First, we tracked depressive symptoms from early pregnancy through multiple stages, providing a detailed understanding of their progression. Second, we conducted a comprehensive assessment of pregnant women’s physical, psychological, and social conditions. Third, we addressed the gaps in the literature by linking these depression trajectories to four adverse perinatal outcomes, enhancing the understanding of antenatal depression’s impact on perinatal health.

This study has several limitations. First, depressive symptoms were self-reported rather than clinically diagnosed, though previous research has confirmed the validity of self-reporting [44]. Second, participants with EPDS scores of 13 or higher were reminded to seek psychiatric diagnosis and treatment, but we did not follow up on referral rates. Third, while controlling for confounding factors, we only accounted for baseline variables significantly linked to adverse outcomes, without considering potential chronic diseases. However, given the low prevalence of chronic diseases in our sample (less than 1%), their impact is likely minimal. Fourth, we did not include any measure of the food environment, which may play a role in maternal mental health and pregnancy outcomes. Additionally, participants lost to follow-up differed from those retained with respect to age, social capital, income, and pregnancy planning, which may have impacted the representativeness of the sample. Future studies should further strengthen follow-up mechanisms to reduce bias and enhance representativeness. Another limitation is the absence of detailed data on antidepressant use. However, previous studies in China and other countries have shown that even when antenatal depression is diagnosed, pregnant women rarely opt for medication due to concerns about potential effects on the fetus and social stigma [45, 46]. Therefore, the impact of this factor on our study is likely to be minimal. Finally, the study was conducted at a single tertiary hospital in China, which may limit the generalizability of the results. Future research should involve more diverse populations and settings to further validate and expand upon our findings.

## Conclusion

This study categorized developmental trajectories of antenatal depressive symptoms into four groups: no depression, persistent subclinical depression, persistent moderate depression, and persistent high depression. The same risk factors across these groups included unplanned pregnancy, low social capital, low psychological resilience, and high perceived stress. Both the persistent moderate and high depression groups were significantly associated with increased risks of GDM and SGA, with the persistent high depression group showing the greatest risk. These findings highlight the critical importance of obstetric healthcare providers addressing key risk factors, particularly for women with moderate to high levels of depressive symptoms, to more effectively manage potential pregnancy complications.

## Supporting information

10.1192/j.eurpsy.2025.10058.sm001 Chen et al. supplementary material Chen et al. supplementary material

## Data Availability

The data supporting the findings of this study are available from the corresponding author upon reasonable request.

## Abbreviations

EPDS: Edinburgh Postnatal Depression Scale
GDM: gestational diabetes mellitus
HDCP: hypertensive disorders complicating pregnancy
PTB: preterm birth
SGA: small for gestational age

## Supplementary material

The supplementary material for this article can be found at http://doi.org/10.1192/j.eurpsy.2025.10058.
