# Supplementary material for: Trajectories of antenatal depression and adverse pregnancy outcomes: A prospective cohort study
Source: Eur Psychiatry. 2025 Jul 21;68(1):e109. doi: 10.1192/j.eurpsy.2025.10058 (PMC12438993; doi:10.1192/j.eurpsy.2025.10058)
Supplement: Chen et al. supplementary material [file S0924933825100588sup001.docx]

**Text S1 Definitions of studied outcomes**

The delivery complications studied included Gestational Diabetes Mellitus (GDM) and Hypertensive Disorders Complicating Pregnancy (HDCP). These complications are defined according to the guidelines issued by the Obstetrics and Gynecology Branch of the Chinese Medical Association. Gestational diabetes is defined as glucose metabolism abnormalities occurring during pregnancy, excluding pre-existing Type I or Type II diabetes. It is diagnosed via an Oral Glucose Tolerance Test (OGTT) conducted between the 24th and 28th weeks of pregnancy, with the diagnostic thresholds being a fasting glucose level of 5.1 mmol/L, 10.0 mmol/L one-hour post-glucose load, and 8.5 mmol/L two hours post-glucose load, with a diagnosis made if any of these thresholds are met or exceeded. Pregnancy-induced hypertension is defined as elevated blood pressure (systolic ≥ 140 mmHg and/or diastolic ≥ 90 mmHg) occurring after the 20th week of pregnancy.^1,2^

The complications extracted in this study include Preterm Birth (PTB) and Small for Gestational Age (SGA). These are defined according to the guidelines on the clinical diagnosis and treatment of preterm labor issued by the Obstetrics and Gynecology Branch of the Chinese Medical Association and the birth size standards for different gestational ages issued by the National Health Commission.^3,4^ Preterm birth is defined as childbirth occurring after the 28th week of pregnancy but before the 37th week, with the infant weighing at least 1000 grams. An infant is considered Small for Gestational Age if their birth weight is below the 10th percentile for their gestational age, with this assessment taking into account the infant's sex and the week of gestation to precisely evaluate growth relative to peers of the same gestational age.^5^

**Table** **S1 Indicators of model fit for the trajectory of prenatal depressive symptoms (N=725)**

| Class | AIC | BIC | aBIC | Entropy | LMRLRT | *p1* | BLRT | *p2* |
| --- | --- | --- | --- | --- | --- | --- | --- | --- |
| 1 | 20951.111 | 20893.214 | 20960.987 |  |  |  |  |  |
| 2 | 19456.275 | 19502.136 | 19470.383 | 0.914 | 1428.537 | <0.001 | -10468.556 | <0.001 |
| 3 | 19008.370 | 19067.990 | 19026.711 | 0.827 | 432 | 0.001 | -9718.137 | 0.001 |
| 4 | 18817.575 | 18890.954 | 18840.149 | 0.843 | 187.315 | <0.001 | -9491.185 | <0.001 |
| 5 | 18757,414 | 18844.551 | 18784.220 | 0.796 | 62.974 | <0.001 | -9392.788 | <0.001 |
| 6 | 18748.878 | 18849.774 | 18779.917 | 0.789 | 13.383 | 0.070 | -9359.707 | 0.010 |

Note: p1 is the corresponding p-value for LMRLRT and p2 is the corresponding p-value for BLR

**Table S2 Number and percentage of potential categories in each model of antenatal depressive symptom development trajectory (N=725)**

| Class | 1 | 2 | 3 | 4 | 5 | 6 |
| --- | --- | --- | --- | --- | --- | --- |
| 1 | 725(100.00%) |  |  |  |  |  |
| 2 | 532(73.38%) | 193(26.62%) |  |  |  |  |
| 3 | 136(18.76%) | 314(43.31%) | 275(37.93%) |  |  |  |
| 4 | 233(32.13%) | 308(42.48%) | 139(19.17%) | 45(6.21%) |  |  |
| 5 | 44(6.07%) | 132(18.21%) | 233(32.14%) | 239(32.97%) | 77(10.62%) |  |
| 6 | 25(3.44%) | 237(32.69%) | 78(10.76%) | 43(5.93%) | 106(14.62%) | 236(32.55%) |

**Table S3 Parameters of the trajectory subgroups of the trajectory model for the development of antenatal depressive symptoms (N=725)**

| Group name | n（%） | Posterior probability | | Intercept | Slope |
| --- | --- | --- | --- | --- | --- |
| No depression group | 233(32.13%) | 0.903 | 5.017 | | -0.403 |
| Persistent subclinical depression group | 308(42.48%) | 0.909 | 7.566 | | 0.087 |
| Persistent moderate depression group | 139(19.17%) | 0.924 | 10.888 | | 0.557 |
| Persistent high depression group | 45(6.21%) | 0.948 | 15.953 | | 0.138 |

**Table S4 Analysis of the trajectory of antenatal depressive symptoms at each stage of the trajectory score and incidence (n=725)**

| Time periods | No depression group（n=233） | | Persistent subclinical depression group （n=308） | | Persistent moderate depression group（n=139） | | Persistent high depression group（n=45） | |
| --- | --- | --- | --- | --- | --- | --- | --- | --- |
| EPDS score |  |  | |  | |  | |  |
| T0 | 6.12±2.42 | 8.36±2.40 | | 11.20±2.90 | | 15.58±2.73 | |  |
| T1 | 3.66±2.34 | 7.15±2.63 | | 11.55±3.20 | | 16.65±2.72 | |  |
| T2 | 3.21±1.99 | 6.91±2.14 | | 11.47±2.86 | | 16.79±2.22 | |  |
| T3 | 3.87±2.18 | 8.28±2.19 | | 13.00±2.75 | | 16.41±2.38 | |  |
| T4 | 4.04±2.69 | 8.34±2.70 | | 13.19±2.95 | | 16.30±3.26 | |  |
| Prevalence |  |  | |  | |  | |  |
| EPDS>9 score (%) |  |  | |  | |  | |  |
| T0 | 9.01% | 34.10% | | 72.66% | | 100% | |  |
| T1 | 0% | 18.83% | | 74.10% | | 100% | |  |
| T2 | 0% | 12.66% | | 80.74% | | 100% | |  |
| T3 | 0% | 27.60% | | 88.4% | | 100% | |  |
| T4 | 3.00% | 35.06% | | 89.62% | | 95.56% | |  |
| EPDS>12 score (%) |  |  | |  | |  | |  |
| T0 | 0% | 1.30% | | 38.84% | | 95.56% | |  |
| T1 | 0% | 2.92% | | 39.57% | | 95.56% | |  |
| T2 | 0% | 1.30% | | 35.56% | | 97.78% | |  |
| T3 | 0% | 4.22% | | 55.56% | | 95.56% | |  |
| T4 | 0% | 5.84% | | 66.67% | | 84.44% | |  |
| EPDS>13 score (%) |  |  | |  | |  | |  |
| T0 | 0% | 0.97% | | 20.86% | | 84.44% | |  |
| T1 | 0% | 0.97% | | 26.67% | | 91.11% | |  |
| T2 | 0% | 0.65% | | 22.22% | | 95.56% | |  |
| T3 | 0% | 0.97% | | 46.67% | | 93.33% | |  |
| T4 | 0% | 0.97% | | 48.89% | | 82.22% | |  |

**Table S5 Repeated Measures ANOVA for Potential Categories of Prenatal Depressive Symptoms (n=725)**

|  | Time | | Latent class | | Time*Latent class | |
| --- | --- | --- | --- | --- | --- | --- |
|  | F | *p* | F | *p* | F | *p* |
| EPDS score | 11.438 | <0.001 | 1973.730 | <0.001 | 16.913 | <0.001 |

**Table S6 Univariate analysis of factors influencing the prenatal depressive symptoms development trajectory(N=725)**

| Variables | Total | The trajectory of prenatal depressive symptoms [M±SD/n (%)] | | | | F/χ**^2^** | *p* |
| --- | --- | --- | --- | --- | --- | --- | --- |
|  |  | No depression group | Persistent subclinical depression group | Persistent moderate depression group | Persistent high depression group |  |  |
|  |  | (n=233) | (n=308) | (n=139) | (n=45) |  |  |
| Age | 32.62±3.72 | 32.41±3.85 | 32.97±3.55 | 32.41±4.06 | 31.96±2.88 | 1.778 | 0.15 |
| Household registration type |  |  |  |  |  | 1.799 | 0.615 |
| City | 411(56.7%) | 127(54.5%) | 182(59.1%) | 75(54.0%) | 27(60.0%) |  |  |
| Rural | 314(43.3%) | 106(45.5%) | 126(40.9%) | 64(46.0%) | 18(40.0%) |  |  |
| Marital status |  |  |  |  |  | 2.872 | 0.326 |
| Married | 699(96.4%) | 227(97.4%) | 298(96.8%) | 132(95%) | 42(93.3%) |  |  |
| Unmarried | 26(3.6%) | 6(2.6%) | 10(3.2%) | 7(5.0%) | 3(6.7%) |  |  |
| Education level |  |  |  |  |  | 10.069 | 0.345 |
| High school and below | 102(14.0%) | 29(12.4%) | 42(13.6%) | 24(17.3%) | 7(15.6%) |  |  |
| Junior college | 187(25.8%) | 52(22.3%) | 86(27.9%) | 38(27.3%) | 11(24.4%) |  |  |
| Undergraduate | 316(43.6%) | 103(44.3%) | 136(44.2%) | 54(38.8%) | 23(51.1%) |  |  |
| Master's degree or above | 120(16.6%) | 49(21.0%) | 44(14.3%) | 23(16.5%) | 4(8.9%) |  |  |
| Monthly family income, yuan |  |  |  |  |  | 7.437 | 0.592 |
| 5,000 and below | 50(6.9%) | 16(7.0%) | 19(6.3%) | 12(8.7%) | 3(6.7%) |  |  |
| 5000~10000 | 226(31.1%) | 73(31.3%) | 88(28.6%) | 47(33.8%) | 18(40.0%) |  |  |
| 10001~20000 | 300(41.4%) | 90(38.6%) | 138(44.8%) | 53(38.1%) | 19(42.2%) |  |  |
| More than 20,000 + | 149(20.6%) | 54(23.2%) | 63(20.5%) | 27(19.4%) | 5(11.1%) |  |  |
| social capital | 46.90±8.21 | 49.14±7.88 | 46.72±7.79 | 44.71±9.01 | 43.40±7.12 | 12.397 | <0.001 |
| pre-pregnancy BMI | 21.80±3.05 | 22.05±3.31 | 21.89±2.81 | 21.26±3.24 | 21.43±2.33 | 2.327 | 0.073 |
| Parity |  |  |  |  |  | 6.769 | 0.08 |
| Primipara | 544(75.0%) | 171(73.4%) | 230(74.7%) | 102(73.4%) | 41(91.1%) |  |  |
| Multipara | 151(25.0%) | 62(26.6%) | 78(25.3%) | 37(26.6%) | 4(8.9%) |  |  |
| gravidity |  |  |  |  |  | 1.781 | 0.939 |
| 1time | 397(54.8%) | 131(56.2%) | 168(54.5%) | 73(52.5%) | 25(55.6%) |  |  |
| 2times | 210(29.0%) | 61(26.2%) | 92(29.9%) | 43(30.9%) | 14(33.1%) |  |  |
| 3times and above | 118(16.2%) | 41(17.6%) | 48(15.6%) | 23(16.5%) | 6(13.3%) |  |  |
| History of adverse pregnancy and delivery |  |  |  |  |  | 0.634 | 0.889 |
| No | 504(69.5%) | 164(70.4%) | 214(69.5%) | 97(69.8%) | 29(64.4%) |  |  |
| Yes | 221(30.5%) | 69(29.6%) | 94(30.5%) | 42(30.2%) | 16(35.6%) |  |  |
| Planned pregnancy |  |  |  |  |  | 14.786 | 0.002 |
| Yes | 566(78.1%) | 201(86.3%) | 233(75.6%) | 101(72.7%) | 31(68.9%) |  |  |
| No | 159(21.9%) | 32(13.7%) | 75(24.4%) | 38(27.3%) | 14(31.1%) |  |  |
| Conception method |  |  |  |  |  | 5.136 | 0.162 |
| Natural conception | 613(84.6%) | 205(88.0%) | 250(81.2%) | 120(86.3%) | 38(84.4%) |  |  |
| Artificial assisted reproduction | 112(15.4%) | 28(12.0%) | 58(18.8%) | 19(13.7%) | 7(15.6%) |  |  |
| History of mental illness |  |  |  |  |  | 35.318 | <0.001 |
| No | 679(93.7%) | 228(97.9%) | 295(95.8%) | 116(83.5%) | 40(88.9%) |  |  |
| Yes | 46(6.3%) | 5(2.1%) | 13(4.2%) | 23(16.5%) | 5(11.1%) |  |  |
| psychological resilience | 26.23±6.48 | 29.09±7.58 | 26.11±5.16 | 22.75±5.18 | 22.94±5.08 | 37.225 | <0.001 |
| perceived stress | 38.59±9.47 | 34.85±11.88 | 40.37±7.36 | 39.16±7.48 | 44.04±7.05 | 22.777 | <0.001 |

Table S7 Univariate analysis between gestational diabetes and baseline characteristics (n=725)

| Item | Gestational diabetes [M±SD/n (%)] | | t/χ^2^ | *p* |
| --- | --- | --- | --- | --- |
|  | No (n=579) | Yes (n=146) |  |  |
| Age, years | 32.56±3.71 | 32.84±3.78 | -0.815 | 0.415 |
| Household |  |  | 0.109 | 0.741 |
| Urban | 330(57.0%) | 81(55.5%) |  |  |
| Rural | 249(43.0%) | 65(44.5%) |  |  |
| Marriage |  |  | 0.014 | 0.906 |
| Married | 558(96.4%) | 141(96.6%) |  |  |
| Unmarried | 21(3.6%) | 5(3.4%) |  |  |
| Education level |  |  | 1.377 | 0.711 |
| ≤High school | 78(13.5%) | 24(16.4%) |  |  |
| Junior college | 151(26.1%) | 36(24.7%) |  |  |
| Undergraduate | 251(43.3%) | 65(44.5%) |  |  |
| ≥Masters | 99(17.1%) | 21(14.4%) |  |  |
| Household income, RMB |  |  | 4.314 | 0.229 |
| ≤5000 | 40(7.0%) | 10(6.8%) |  |  |
| 5000-10000 | 171(28.9%) | 55(37.5%) |  |  |
| 10001-20000 | 249(43.6%) | 51(35.4%) |  |  |
| ≥20000 | 119(20.5%) | 30(20.1%) |  |  |
| Social capital score | 47.24±7.84 | 45.57±9.47 | 1.969 | 0.050 |
| Pre-pregnancy BMI, kg/m^2^ | 21.60±2.92 | 22.59±3.39 | -3.533 | <0.001 |
| Parity |  |  | 2.541 | 0.111 |
| Primigravida | 427(73.7%) | 117(80.1%) |  |  |
| Menstruation | 152(26.3%) | 29(19.9%) |  |  |
| Number of pregnancies |  |  |  |  |
| Once | 309(53.4%) | 88(60.3%) | 2.531 | 0.282 |
| Twice | 171(29.5%) | 39(26.7%) |  |  |
| ≥3 times | 99(17.1%)) | 19(13.0%) |  |  |
| Adverse Maternity History |  |  | 1.713 | 0.191 |
| Yes | 396(68.4%) | 108(74.0%) |  |  |
| No | 183(31.6%) | 38(26.0%) |  |  |
| Planned pregnancy |  |  | 0.048 | 0.826 |
| Yes | 453(78.2%) | 113(77.4%) |  |  |
| No | 126(21.8%) | 33(22.6%) |  |  |
| Method of conception |  |  | 2.728 | 0.099 |
| Natural pregnancy | 496(85.7%) | 117(80.1%) |  |  |
| Artificial assisted reproduction | 83(14.3%) | 29(19.9%) |  |  |
| History of mental illness |  |  | 1.081 | 0.299 |
| Yes | 545(94.1%) | 134(91.8%) |  |  |
| No | 34(5.9%) | 12(8.2%) |  |  |
| Psychological resilience | 26.45±6.50 | 25.34±6.35 | 1.864 | 0.063 |
| Perceived Stress | 38.43±9.57 | 39.25±9.07 | -0.940 | 0.347 |

Table S8 Univariate analysis between gestational hypertension and baseline characteristics (n=725)

| Item | Gestational hypertension [M±SD/n (%)] | | t/χ^2^ | *p* |
| --- | --- | --- | --- | --- |
|  | No (n=680) | Yes (n=45) |  |  |
| Age, years | 32.58±3.70 | 33.13±4.05 | -0.959 | 0.338 |
| Household |  |  | 0.025 | 0.874 |
| Urban | 386(56.8%) | 25(55.6%) |  |  |
| Rural | 294(43.2%) | 20(44.4%) |  |  |
| Marriage |  |  | 1.785 | 0.182 |
| Married | 654(96.2%) | 45(100.0%) |  |  |
| Unmarried | 26(3.8%) | 0(0.0%) |  |  |
| Education level |  |  | 0.811 | 0.847 |
| ≤High school | 94(13.8%) | 8(17.8%) |  |  |
| Junior college | 176(25.9%) | 11(24.4%) |  |  |
| Undergraduate | 296(43.5%) | 20(44.4%) |  |  |
| ≥Masters | 114(16.8%) | 6(13.4%) |  |  |
| Household income, RMB |  |  | 3.800 | 0.284 |
| ≤5000 | 49(7.2%) | 1(2.2%) |  |  |
| 5000-10000 | 207(30.4%) | 19(42.2%) |  |  |
| 10001-20000 | 284(41.8%) | 16(35.6%) |  |  |
| ≥20000 | 140(20.6%) | 9(20.0%) |  |  |
| Social capital score | 49.69±8.47 | 46.72±8.17 | 2.356 | 0.019 |
| Pre-pregnancy BMI, kg/m^2^ | 21.70±3.01 | 23.27±3.24 | -3.378 | <0.001 |
| Parity |  |  | 0.394 | 0.530 |
| Primigravida | 512(75.3%) | 32(71.1%) |  |  |
| Menstruation | 168(24.7%) | 13(28.9%) |  |  |
| Number of pregnancies |  |  | 0.337 | 0.845 |
| Once | 372(54.7%) | 25(55.6%) |  |  |
| Twice | 196(28.8%) | 14(31.1%) |  |  |
| ≥3 times | 112(16.5%) | 6(13.3%) |  |  |
| Adverse Maternity History |  |  | 0.058 | 0.810 |
| Yes | 472(69.4%) | 32(71.1%) |  |  |
| No | 208(30.6%) | 13(28.9%) |  |  |
| Planned pregnancy |  |  | 0.002 | 0.961 |
| Yes | 531(78.1%) | 35(77.28%) |  |  |
| No | 149(21.9%) | 10(22.2%) |  |  |
| Method of conception |  |  | 0.164 | 0.685 |
| Natural pregnancy | 574(84.4%) | 39(86.7%) |  |  |
| Artificial assisted reproduction | 106(15.6%) | 6(13.3%) |  |  |
| History of mental illness |  |  | 0.008 | 0.927 |
| Yes | 637(93.7%) | 42(93.3%) |  |  |
| No | 43(6.3%) | 3(6.7%) |  |  |
| Psychological resilience | 26.28±6.47 | 25.47±6.75 | 0.815 | 0.415 |
| Perceived Stress | 33.97±11.51 | 38.90±9.25 | -3.407 | <0.001 |

Table S9 Univariate analysis between of preterm birth and baseline characteristics (n=725)

| Item | Preterm birth [M±SD/n (%)] | | t/χ^2^ | *p* |
| --- | --- | --- | --- | --- |
|  | No (n=613) | Yes (n=112) |  |  |
| Age, years | 32.52±3.67 | 33.17±3.98 | -1.708 | 0.088 |
| Household |  |  | 0.096 | 0.757 |
| Urban | 349(56.9%) | 62(55.4%) |  |  |
| Rural | 264(43.1%) | 50(44.6%) |  |  |
| Marriage |  |  | 0.316 | 0.574 |
| Married | 590(96.2%) | 109(97.3%) |  |  |
| Unmarried | 23(3.8%) | 3(2.7%) |  |  |
| Education level |  |  | 3.937 | 0.268 |
| ≤High school | 83(13.5%) | 19(17.0%) |  |  |
| Junior college | 152(24.8%) | 35(31.2%) |  |  |
| Undergraduate | 273(44.5%) | 43(38.4%) |  |  |
| ≥Masters | 105(17.2%) | 15(13.4%) |  |  |
| Household income, RMB |  |  | 0.371 | 0.946 |
| ≤5000 | 42(6.9%) | 8(7.1%) |  |  |
| 5000-10000 | 189(30.8%) | 37(33.0%) |  |  |
| 10001-20000 | 254(41.4%) | 46(41.1%) |  |  |
| ≥20000 | 128(20.9%) | 21(18.8%) |  |  |
| Social capital score | 47.21±8.17 | 45.21±8.28 | 2.386 | 0.017 |
| Pre-pregnancy BMI, kg/m^2^ | 21.70±2.99 | 22.30±3.32 | -1.933 | 0.027 |
| Parity |  |  | 8.170 | 0.004 |
| Primigravida | 472(77.0%) | 72(64.3%) |  |  |
| Menstruation | 141(23.0%) | 40(35.7%) |  |  |
| Number of pregnancies |  |  | 5.763 | 0.056 |
| Once | 347(56.6%) | 50(44.6%) |  |  |
| Twice | 172(28.1%) | 38(33.9%) |  |  |
| ≥3 times | 94(15.3%) | 24(21.5%) |  |  |
| Adverse Maternity History |  |  | 2.345 | 0.126 |
| Yes | 433(70.6%) | 71(63.4%) |  |  |
| No | 180(29.4%) | 41(36.6%) |  |  |
| Planned pregnancy |  |  | 1.823 | 0.177 |
| Yes | 484(79.0%) | 82(73.2%) |  |  |
| No | 129(21.0%) | 30(18.9%) |  |  |
| Method of conception |  |  | 1.784 | 0.182 |
| Natural pregnancy | 523(85.3%) | 90(80.4%) |  |  |
| Artificial assisted reproduction | 90(14.7%) | 22(19.6%) |  |  |
| History of mental illness |  |  | 0.142 | 0.706 |
| Yes | 575(93.8%) | 104(92.9%) |  |  |
| No | 38(6.2%) | 8(7.1%) |  |  |
| Psychological resilience | 26.21±6.58 | 26.31±5.94 | -0.147 | 0.883 |
| Perceived Stress | 38.74±8.87 | 37.82±12.26 | 0.942 | 0.173 |

Table S10 Univariate analysis between Small-for-gestational-age infants with baseline characteristics (n=725)

| Item | SGA [M±SD/n (%)] | | t/χ^2^ | *p* |
| --- | --- | --- | --- | --- |
|  | No (n=658) | Yes (n=67) |  |  |
| Age, years | 32.62±3.75 | 32.61±3.44 | 0.014 | 0.989 |
| Household |  |  | 0.000 | 0.996 |
| Urban | 373(56.7%) | 38(56.7%) |  |  |
| Rural | 285(43.3%) | 29(43.3%) |  |  |
| Marriage |  |  | 0.077 | 0.781 |
| Married | 634(96.4%) | 65(97.0%) |  |  |
| Unmarried | 24(3.6%) | 2(3.0%) |  |  |
| Education level |  |  | 3.197 | 0.362 |
| ≤High school | 95(14.4%) | 7(10.4%) |  |  |
| Junior college | 172(26.1%) | 15(22.4%) |  |  |
| Undergraduate | 280(42.6%) | 36(53.7%) |  |  |
| ≥Masters | 111(16.9%) | 9(13.4%) |  |  |
| Household income, RMB |  |  | 1.651 | 0.648 |
| ≤5000 | 44(6.7%) | 6(9.0%) |  |  |
| 5000-10000 | 209(31.8%) | 17(25.4%) |  |  |
| 10001-20000 | 272(41.3%) | 28(41.8%) |  |  |
| ≥20000 | 133(20.2%) | 16(23.9%) |  |  |
| Social capital score | 47.02±8.31 | 45.76±7.18 | 1.195 | 0.233 |
| Pre-pregnancy BMI, kg/m^2^ | 21.84±3.05 | 21.31±2.99 | 1.383 | 0.167 |
| Parity |  |  | 1.961 | 0.161 |
| Primigravida | 489(74.3%) | 55(82.1%) |  |  |
| Menstruation | 169(25.7%) | 12(17.9%) |  |  |
| Number of pregnancies |  |  | 4.721 | 0.094 |
| Once | 353(53.6%) | 44(65.7%) |  |  |
| Twice | 198(30.1%) | 12(17.9%) |  |  |
| ≥3 times | 107(16.3%) | 11(16.4%) |  |  |
| Adverse Maternity History |  |  | 0.459 | 0.500 |
| Yes | 455(69.1%) | 49(73.1%) |  |  |
| No | 203(30.9%) | 18(26.9%) |  |  |
| Planned pregnancy |  |  | 1.311 | 0.252 |
| Yes | 510(77.5%) | 56(83.6%) |  |  |
| No | 148(22.5%) | 11(16.4%) |  |  |
| Method of conception |  |  | 0.053 | 0.818 |
| Natural pregnancy | 577(84.7%) | 56(83.6%) |  |  |
| Artificial assisted reproduction | 101(15.3%) | 11(16.4%) |  |  |
| History of mental illness |  |  | 1.402 | 0.302 |
| Yes | 614(93.3%) | 65(97.0%) |  |  |
| No | 44(6.7%) | 2(3.0%) |  |  |
| Psychological resilience | 26.34±6.64 | 25.20±4.61 | 1.837 | 0.069 |
| Perceived Stress | 38.49±9.48 | 39.58±9.33 | -0.896 | 0.371 |

**References**

1. Department of Obstetrics and Gynecology, Chinese Society of Obstetrics and Gynecology, Chinese Society of Perinatal Medicine, Professional Committee of Pregnancy and Diabetes Mellitus, Chinese Maternal and Child Health Association. Guidelines for diagnosis and treatment of hyperglycemia in pregnancy (2022) [Part 1]. *Chin J Obstet Gynecol*. 2022;57(1):3-12. (In Chinese)

2. Pregnancy-induced Hypertension Disease Group, Chinese Society of Obstetrics and Gynecology. Guidelines for diagnosis and treatment of hypertensive diseases in pregnancy (2020). *Chin J Obstet Gynecol*. 2020;55(4):227-238. (In Chinese)

3. National Health Commission of the People's Republic of China. Growth evaluation criteria for newborns of different gestational ages at birth. 2022;(11; 11.020):10. (In Chinese)

4. Hu Y. Clinical diagnosis and treatment guidelines for premature birth (2014). *Chin J Obstet Gynecol*. 2014;(7):481-485. (In Chinese)

5. Deng Z, Luo XQ. Growth and development of infants small for gestational age and research progress. *Chin Clin J Pract Pediatr*. 2019;34(14):1105-1108. (In Chinese)
